# Multifaceted Anticancer Activity of Flavanone/Chromanone Intermediates for Five-Membered Heterocyclic Derivatives: Targeting Oxidative Stress, Apoptosis, and MAPK Signaling in Colorectal Cancer

**DOI:** 10.3390/molecules31030534

**Published:** 2026-02-03

**Authors:** Pawel Hikisz, Angelika A. Adamus-Grabicka, Elzbieta Budzisz

**Affiliations:** 1 Department of Oncobiology and Epigenetics, Institute of Biophysics, Faculty of Biology and Environmental Protection, University of Lodz, Pomorska 141/143, 90-236 Lodz, Poland; pawel.hikisz@biol.uni.lodz.pl; 2 Department of Bioinorganic Chemistry, Faculty of Pharmacy, Medical University of Lodz, Muszynskiego 1, 90-151 Lodz, Poland; 3 Department of the Chemistry of Cosmetic Raw Materials, Medical University of Lodz, 90-151 Lodz, Poland

**Keywords:** flavonoid derivatives, pyrazolines, five-membered scaffolds, anticancer properties, Nrf2, MAPK pathway, apoptosis, reactive oxygen species, genotoxicity, colon carcinoma

## Abstract

This study explores the multifaceted anticancer mechanisms of flavanone analogues and spiropyrazoline condensed with flavanone ring against colorectal cancer (CRC) cell lines. Five-membered heteroaromatic scaffolds, in particular, have gained prominence in medicinal chemistry as they offer enhanced metabolic stability, solubility and bioavailability, crucial factors in developing effective drugs. Building upon previous findings, we investigated three lead derivatives (**1**, **3**, and **5**) with potent antiproliferative activity (IC_50_ < 35 μM). The compounds induced pronounced oxidative stress, evidenced by increased lipid peroxidation and reduced membrane fluidity, primarily within the hydrophobic layers of cell membranes. Preincubation with the antioxidant N-acetylcysteine (NAC) significantly attenuated these effects, confirming the pivotal role of reactive oxygen species (ROS) in their cytotoxicity. Mechanistic studies revealed that the derivatives triggered intrinsic apoptosis, characterized by the cleavage of PARP and the activation of caspase-9 and caspase-3. Furthermore, the compounds modulated key signaling pathways involved in cell survival and proliferation. Specifically, they inhibited the pro-oncogenic ERK1/2 MAPK pathway while inducing cell line-dependent alterations in p38 and JNK activity. Concurrently, all derivatives reduced the level of the transcription factor Nrf2, a master regulator of antioxidant defense and a mediator of chemoresistance in CRC. Collectively, these findings indicate that flavanone/chromanone derivatives exert their anticancer activity through a synergistic mechanism involving ROS generation, disruption of redox homeostasis, inhibition of Nrf2 signaling, and modulation of MAPK-dependent apoptotic pathways. These results highlight the therapeutic potential of flavanone-based compounds and their spiropyrazoline analogues as multifunctional anticancer agents targeting oxidative stress and survival signaling in colorectal cancer.

## 1. Introduction

Cancer is intimately linked to increasing life expectancy and the progressive aging of societies in high- and middle-income countries. For several decades, epidemiological data have consistently indicated an increase in the incidence and mortality rates of colorectal cancer (CRC) worldwide [1].

CRC is currently one of the most common malignancies globally and is the second leading cause of cancer-related deaths. Unfortunately, future projections regarding the incidence of this type of cancer are alarming, with the number of new cases and associated deaths expected to increase in the coming years. This increase mainly affects countries undergoing economic transformation. Importantly, a concerning rise in incidence is also being observed among younger adults (under 50 years of age), even in developed countries [2].

Long-term epidemiological studies indicate that environmental factors are among the most important risk factors in the etiology of colorectal cancer (CRC). Overall lifestyle, nutrition, and diet are particularly critical contributors. An unhealthy lifestyle, frequently associated with excessive alcohol and tobacco consumption or a poor diet rich in “empty” calories but lacking in essential nutrients, correlates strongly with an increased incidence of CRC [3].

Furthermore, such an unhygienic lifestyle often involves chronic stress, lack of physical activity, widespread antibiotic use that alters the gut microflora, smoking, and obesity. These factors can lead to metabolic dysfunction and related inflammation, chronic oxidative stress, impaired gut microflora, and ultimately, a compromised intestinal barrier integrity [2,3]. It is worth paying attention to your diet, especially the consumption of red meat. There are several reasons why red meat can be harmful to your health. Red meat contains saturated fat, which is fat that is solid at room temperature. Although the body needs fat, excess fat can increase the risk of heart disease, stroke, and lead to obesity, which is a risk factor for cancer. Another important factor is the processing of the red meat such as through smoking or salting or through chemical preservatives such as nitrates and nitrites. Eating processed meats increases the risk of colorectal cancer. During the cooking of red meat, especially at high temperatures, harmful compounds are released. According to the National Cancer Institute (NCI), these substances are heterocyclic amines (HCAs) and polycyclic aromatic hydrocarbons (PAHs). Research shows that HCAs and PAHs can alter DNA, possibly increasing colorectal cancer risk [4,5,6].

Despite the significant contribution of environmental factors to the incidence of colorectal cancer (CRC), genetic factors also play a significant role in its pathogenesis. It is widely accepted that hereditary or spontaneous mutations in specific genes, combined with environmental exposure, serve as potent initiators of carcinogenesis.

It should be noted that the development of CRC is primarily driven by somatic mutations (those arising de novo during an individual’s lifetime). However, some patients harbor germline mutations—inherited from the parents and present from birth—in genes critical to tumor initiation and progression, which significantly increases their CRC risk. Key genes implicated in these mutations include the tumor suppressor genes *APC* (Adenomatous Polyposis Coli) and *TP53* (Tumor suppressor p53), as well as the oncogene *KRAS* (Kirsten rat sarcoma virus).

Furthermore, the literature highlights the significant involvement of hereditary syndromes in CRC oncogenesis, such as Lynch syndrome, familial adenomatous polyposis (FAP), and MUTYH-associated polyposis (MAP), which involves the *MUTYH* DNA glycosylase gene (encoding a protein crucial for DNA repair) [7,8].

Modern medicine continually faces the challenge of discovering, synthesizing, and ensuring the appropriate physicochemical stability of new compounds with desirable pharmacological activity. This pursuit is crucial not only for finding safer drugs for patients but also in the context of increasing cancer resistance to currently used chemotherapeutic agents. Although commonly employed drugs often demonstrate satisfactory anticancer activity, they unfortunately suffer from high systemic cytotoxicity, which significantly limits their widespread use. The persistent problem of low specificity of action in commercially available chemotherapeutic agents has thus provided an impetus for intensified research into new compounds that combine attractive anticancer properties with low systemic toxicity [9,10].

Flavanones (2-phenyl-2,3-dihydro-4*H*-chromen-4-one), a subgroup of natural plant polyphenols known as flavonoids, are under intensive investigation for their potential in cancer treatment and prevention, whether as standalone chemotherapeutic agents or as adjuncts to conventional chemotherapy. These compounds are essential components of the daily diet and medicinal plant materials [11,12,13,14].

Numerous publications indicate that flavanones, such as naringenin [15] (found in grapefruits) and hesperidin [16] (found in oranges), exhibit multifaceted biological effects that can be utilized to combat cancer cells. The diversity of their cellular actions is compelling evidence of their broad spectrum of anticancer activity [17,18]. Research suggests that flavanones may affect cancer through complex molecular mechanisms, including induction of apoptosis and activation of proapoptotic signaling pathways, modulation of the MAPK (Mitogen-activated protein kinase) pathway, modulating the expression of genes encoding proteins from the Bcl-2 family, and inhibiting the cell cycle and proliferation of cancer cells [11,12].

Importantly, available research indicates that flavanones may be promising agents in combating multidrug resistance (MDR), one of the main problems in modern chemotherapy. They can act synergistically with conventional cytostatics, increasing their effectiveness and potentially reducing toxicity to healthy cells, thus serving as “sensitizers” for anticancer therapy [19,20].

Flavanones hold great promise in oncology, but their role as full-fledged chemotherapeutic agents requires further, extensive clinical trials in humans to fully assess their efficacy, safety, and bioavailability. It is noteworthy that while current scientific evidence of their biological activity is predominantly derived from laboratory (in vitro) studies, flavanones are a promising class of materials for new drug development because they are characterized by low systemic toxicity and demonstrate a relatively high ease of chemical modification and synthesis, for example, of complex metal complexes [21,22,23].

Five-membered heterocyclic compounds, such as pyrazoles, play a significant role in medicinal chemistry. Representatives of these compounds form the basis of numerous drugs with diverse therapeutic activities, including anticancer, antimicrobial, antiviral, anti-inflammatory, analgesic, and antidiabetic properties [24]. The unique physicochemical properties and biological effects of five-membered heterocycles have made them key structural motifs in many clinically effective drugs. Our team have long been investigating the biological properties of pyrazolines fused to a chromone ring substituted with various functional groups [25,26]. Due to the combination of the pyrazoline scaffold containing nitrogen atoms with compounds with flavone scaffold, the biological potential of these compounds is greater [27].

**Figure 1:**
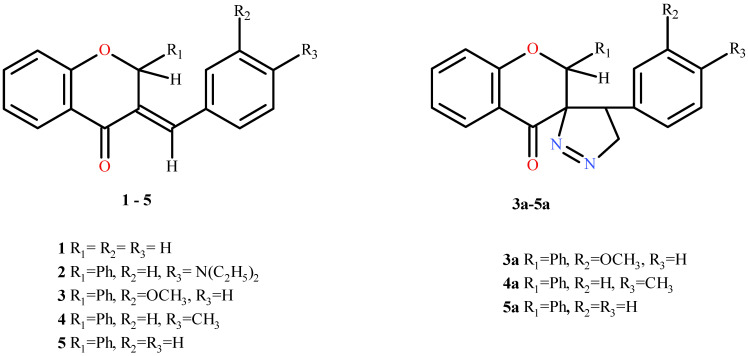
The structures of tested compounds.

This publication serves as a continuation and logical scientific expansion of our previous studies [28] on the molecular mechanisms underpinning the anticancer activity of flavanone/chromanone derivatives. The same five cancer cell lines were utilized in both studies. The physicochemical properties and synthesis of the complete set of derivatives were detailed in our prior work [26]. In our previous studies, we reported the synthesis, physicochemical characterization, and preliminary cytotoxic evaluation of a series of flavanone/chromanone derivatives and their five-membered heterocyclic analogues [26,28]. The present manuscript builds directly upon these findings. However, for the convenience and clarity of the current article, the structural formulas of the selected derivatives used in the present study are included in Figure 1. Building upon our previous findings, which established the cytotoxicity and general pro-oxidant potential of these derivatives [28], the present study aims to provide a comprehensive mechanistic evaluation. While our earlier work focused on mitochondrial potential and basic oxidative markers, here, we extend the investigation to include the direct impact of ROS (reactive oxygen species) generation on the physicochemical integrity of cell membranes, specifically lipid peroxidation and hydrophobic layer fluidity. Furthermore, we delineate the downstream signaling pathways by linking oxidative stress to the caspase-9/caspase-3/PARP (Poly(ADP-ribose) Polymerase) apoptotic cascade and the suppression of the Nrf2-mediated (Nuclear factor erythroid 2-related factor 2) antioxidant defense system. Additionally, this study offers the first detailed analysis of cell-line-specific MAPK (Mitogen-Activated Protein Kinase) (ERK/JNK/p38) modulation in response to these compounds, thereby providing a deeper understanding of their complex mode of action in cancer cells.

For this study, five colorectal cancer cell lines were selected, namely, HCT116, SW620, LoVo, Caco-2, and HT-29, in order to broadly reflect the molecular and biological heterogeneity of this type of cancer. These lines differ significantly in the status of key oncogenes and tumor suppressor genes, including *KRAS*, *BRAF*, and *TP53*, as well as in the activity levels of the MAPK and PI3K/AKT pathways, which are closely associated with the regulation of oxidative stress, proliferation, and apoptosis.

HCT116 and LoVo cells carry activating *KRAS* mutations and exhibit high pro-proliferative pathway activity along with elevated basal oxidative stress. SW620, derived from a lymph node metastasis, represents a more aggressive tumor phenotype and allows for the assessment of the studied compounds in the context of tumor progression and metastatic potential. In contrast, HT-29 is a *KRAS* wild-type line but harbors the *BRAF* V600E mutation, representing a distinct molecular subtype of colorectal cancer with dysregulated MAPK signaling and a different response to oxidative stress. Caco-2 cells, on the other hand, display a more differentiated, enterocyte-like phenotype and a relatively stable genetic profile, allowing for the analysis of responses in cells with a higher degree of differentiation.

This carefully selected panel of cell lines enables the evaluation of whether the observed effects of the tested compounds—particularly the induction of oxidative stress, modulation of MAPK pathways, and activation of apoptosis—are maintained across different molecular and biological contexts, rather than being limited to a single colorectal cancer subtype. Such an approach enhances the biological relevance of the results and provides a better understanding of the influence of genetic background on the sensitivity of cancer cells.

We emphasize that this publication focuses on the detailed biological studies of three flavanone/chromanone derivatives, designated as derivative **1**, **3**, and **5** in the previous work [28]. All experiments conducted here are a direct continuation of prior research and constitute a complementary, unified approach. The selection of these specific compounds was based on preliminary screening results from MTT assays (using 4,5-dimethylthiazol-2-yl)-2,5-diphenyltetrazolium bromide) and the IC_50_ concentrations calculated previously [28]. Compounds **1**, **3**, and **5** were chosen due to their promising antiproliferative activity, with an IC_50_ threshold of 35 μM established as the cutoff for considering compounds as potentially biologically active chemotherapeutic candidates. Importantly, these compounds represent key synthetic intermediates used for the preparation of their corresponding derivatives based on a five-membered heterocyclic ring, including spiropyrazoline analogues, as described in our earlier synthetic and biological studies [26,28]. While their basic antiproliferative properties have already been established, the present work focuses on a comprehensive mechanistic analysis of their anticancer activity, including oxidative stress induction, modulation of MAPK signaling pathways, apoptosis activation, and inhibition of the Nrf2-dependent antioxidant response.

Thus, the current study constitutes a logical continuation of our previous research and provides novel mechanistic insights that were not addressed in earlier publications.

## 2. In Vitro Effects of Flavanone/Chromanone Derivatives on PARP Degradation

Poly (ADP-ribose) polymerase (PARP) is a key enzyme in DNA damage repair, and its proper function is critical for maintaining genomic stability. Dysfunction, such as mutations or excessive activity, can promote cancer progression. This is because cancer cells, characterized by rapid proliferation, often depend on these enzymes to repair the DNA damage that results from their fast growth and division. Consequently, PARP inhibition and degradation are considered a promising therapeutic strategy for inducing effective cancer cell death [29,30].

**Figure 2:**
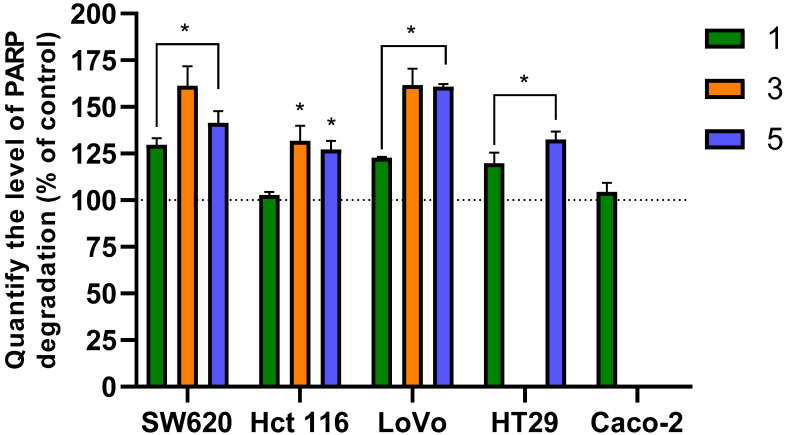
Measurement of PARP degradation in HCT 116, SW620, LoVo, Caco-2 and HT-29 cells exposed to compound **1**, **3** and **5**. Cells were incubated with derivatives for 24 h at concentrations of the calculated IC_50_. The results represent mean ± SD of the data from 3 individual experiments, untreated control cells arbitrarily taken as 100%, * *p* < 0.05 vs. control.

A 24 h exposure of colon cancer cells to the tested flavanone/chromanone derivatives resulted in significant PARP degradation (Figure 2). Analogue **1** was an exception, showing no statistically significant changes in the HCT 116 and Caco-2 cell lines. For the remaining cell lines—namely, SW620, LoVo, and HT-29—PARP degradation was approximately 20–30% relative to control cells. Notably, derivatives **3** and **5** induced significantly greater degradation, ranging from ~40 to 60% depending on the cell line.

Analysis of the results indicates that the HCT116 and HT-29 lines were slightly less sensitive to PARP inhibition than the SW620 and LoVo lines. In these less sensitive lines, PARP inhibition for the most active derivatives peaked at ~30% compared to untreated control cells. However, this still represents a substantial level of PARP degradation induced by the tested derivatives. In contrast, the most significant changes were observed in the SW620 and LoVo lines, where derivative **3** induced degradation levels up to ~60% relative to the control. The degradation of the PARP enzyme by caspases is a key event in the apoptotic pathway, leading to programmed cell death and preventing the repair of damaged DNA.

Poly (ADP-ribose) polymerase (PARP) is a key enzyme in DNA repair and is often overexpressed in various cancers, including colorectal cancer (CRC). This overexpression can promote resistance to apoptosis, making PARP a compelling target for enhancing the efficacy of existing therapies. The inhibition and degradation of PARP using natural or synthetic flavonoids is an area of growing interest in anticancer therapy [31]. PARP degradation is a critical step in apoptosis. Normally involved in DNA repair, the enzyme is cleaved by caspases, which inactivates the repair process in cancer cells and leads to their death. The presence of the degraded 89-kDa PARP fragment is a widely accepted indicator of apoptosis, distinguishing it from necrosis [32]. The significant PARP degradation observed in colon cancer cells exposed to the flavanone/chromanone analogues analyzed in our study indicates their potent proapoptotic properties. These findings are consistent with the prior literature. Studies indicate that synthetic flavanones, in the course of their proprioceptive activity, lead to the degradation of the PARP enzyme in cancer cells [33,34]. For instance, studies have shown that synthetic flavanones can enhance the effectiveness of TNF-related apoptosis-inducing ligand (TRAIL) in inducing apoptosis in cancer cells [31]. This apoptosis is mediated by proapoptotic pathways linked to PARP activity, supporting the hypothesis that flavanones can indirectly inhibit PARP function in CRC. The synergistic effect of combining flavanones with agents like TRAIL suggests that PARP may be a functional target for these compounds [31]. Furthermore, the natural flavonoid naringenin has been shown to activate apoptotic processes through pathways involving PARP cleavage, highlighting its potential as a therapeutic agent for CRC [35]. Similarly, flavonoids from plants like *Daphne genkwa* exhibit significant antiproliferative effects and activate apoptosis in colon cancer cells [36]. The link between PARP overexpression and tumor progression is well-established, with PARP-1 overexpression frequently associated with advanced CRC stages [37]. This makes PARP an appropriate target for flavonoid-based therapies. Some flavonoids can inhibit excessive PARP activation, which reduces tumor aggressiveness by limiting the DNA repair capacity of cancer cells [38]. The induction of programmed cell death (PCD) combined with PARP inhibition is a critical strategy to enhance the efficacy of chemotherapy, especially in cancers with BRCA deficiencies or other mutations in DNA repair pathways [35,39].

## 3. Lipid Peroxidation and Changes in Cell Membrane Fluidity

Reactive oxygen species (ROS) can initiate lipid peroxidation, a chain reaction in which the unsaturated fatty acids of cell membranes are oxidized. Similarly to how they affect healthy cells, free radicals damage cancer cell membranes, and lipid peroxidation often leads to membrane stiffening. This stiffening impairs the movement of essential proteins and lipids, disrupting membrane integrity and the transport of ions and other substances. In severe cases, this damage can lead to programmed cell death (apoptosis) [40,41].

**Figure 3:**
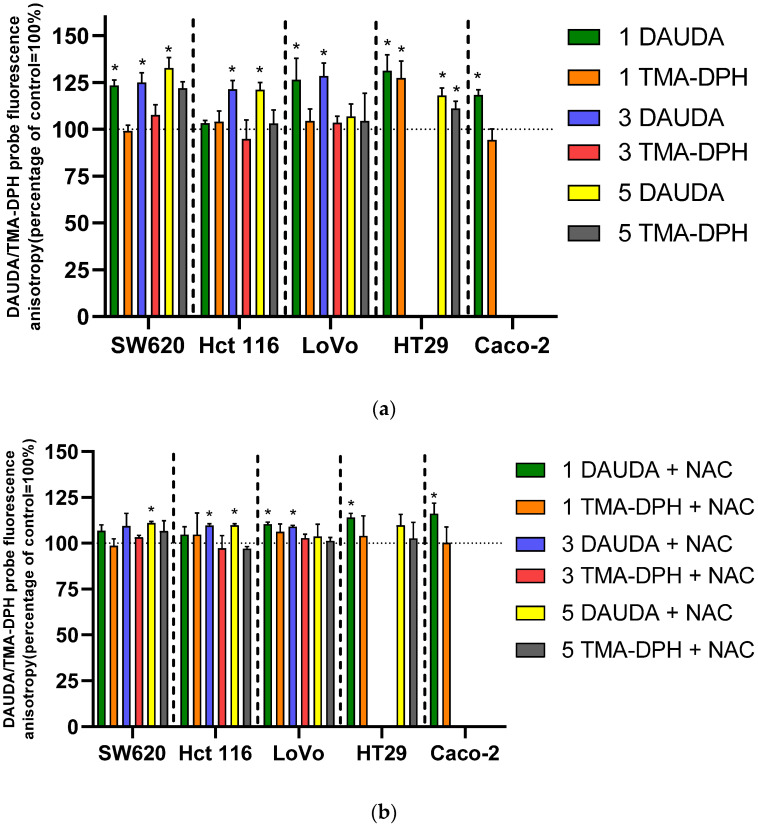
(**a**) Fluorescence anisotropy of DAUDA and TMA-DPH label in HCT 116, SW620, LoVo, Caco-2 and HT-29 cells exposed to compound **1**, **3** and **5**. Results represent mean ± SD of data from three individual experiments, each performed with at least six repeats; * *p* < 0.05 versus control. (**b**) Fluorescence anisotropy of DAUDA and TMA-DPH label in HCT 116, SW620, LoVo, Caco-2 and HT-29 cells exposed to compound **1**, **3** and **5** after 1 h preincubation with NAC. Results represent mean ± SD of data from three individual experiments, each performed with at least six repeats; * *p* < 0.05 versus control.

Based on our prior findings and the potent prooxidant properties of the tested derivatives [8], we investigated changes in the fluidity of cancer cell membranes following a 24 h exposure to the derivatives at their IC_50_ concentrations. To probe different depths of the lipid bilayer, we used two fluorescent probes: 1-(4-(trimethylamino) phenyl)-6-phenylhexa-1,3,5-triene (TMA-DPH) and 11-(Dansylamino)undecanoic acid (DAUDA). TMA-DPH incorporates into the outer, hydrophilic surface of the membrane, while DAUDA penetrates the deeper, hydrophobic region. To verify the involvement of ROS in lipid peroxidation, we also analyzed membrane fluidity after a one-hour preincubation with N-acetylcysteine (NAC). The results of changes in membrane fluidity are presented in Figure 3a (without NAC antioxidant) and Figure 3b (with NAC antioxidant).

The tested compounds significantly affected the fluidity of cancer cell membranes, causing stiffening. We observed notably greater changes in the hydrophobic portions of the unsaturated fatty acid chains (DAUDA probe) compared to the outer, hydrophilic heads (TMA-DPH probe). Statistically significant changes in the outer hydrophilic regions were only observed in the SW620 and HT29 cell lines. In SW620 cells, derivative **5** caused a decrease in membrane fluidity of approximately 20% relative to control cells. This same derivative had a slightly weaker effect on the HT29 line, causing a decrease of about 10%. However, for the HT29 line, derivative **1** caused a significant increase in membrane stiffness of approximately 25%. Significantly greater changes were observed with the DAUDA probe, which targets the deeper hydrophobic layers. The tested derivatives caused substantial changes in membrane fluidity in all cancer cell lines, with an average increase in stiffness within the inner hydrophobic layers ranging from approximately 20–35%. The SW620 line remained the most sensitive, with a decrease in membrane fluidity of approximately 25–35% for all three tested derivatives. In the other cell lines, the decrease in fluidity was slightly less, ranging from 20 to 25 observed no statistically significant changes in isolated cases.

Preincubation with NAC significantly altered the activity of the derivatives, reducing membrane lipid peroxidation and changes in fluidity. For the outer hydrophilic region (TMA-DPH), no statistically significant changes were observed in the presence of NAC. More importantly, the one-hour preincubation with NAC inhibited changes in membrane fluidity in the hydrophilic region of SW620 and HT29 cells for derivatives **1** and **5**.

The antioxidant also significantly inhibited lipid peroxidation and changes in fluidity in the inner hydrophobic layers of the cell membranes. A one-hour preincubation with NAC reduced the increase in membrane stiffness by an average of 15–20%, depending on the cell line. This indicates a significant contribution of the ROS generated by the tested derivatives to the observed changes in cell membrane fluidity and lipid peroxidation.

Flavonoids are a complex group of compounds primarily known for their potent antioxidant properties, which neutralize free radicals. However, under specific conditions, they can also exhibit prooxidant effects, acting as a “double-edged sword” in oxidative stress management [42,43,44]. The balance between these opposing effects depends largely on factors such as concentration, molecular structure, and the presence of environmental factors like metal ions (e.g., iron or copper) that catalyze redox reactions [45,46]. In cancer cells, which often have elevated ROS levels and compromised antioxidant systems, the additional ROS generated by flavonoids can exceed the cell’s tolerance threshold. This leads to oxidative stress, which damages key cellular components—proteins, lipids, and DNA—and ultimately induces apoptosis [45,47]. Our previous studies with the chromanone derivatives also confirmed their significant prooxidant properties [28].

Lipids are a primary cellular target for ROS. ROS-induced lipid peroxidation plays a significant role in the pathophysiology of cancer and is a key factor in many cancer treatments [48,49]. As Tai et al. [50] explained, oxidative stress-induced lipid damage leads to structural disorders in cell membranes, directly affecting their fluidity, a property critical for many cellular processes [50]. Furthermore, mitochondrial ROS, particularly hydroxyl radicals, can induce lipid peroxidation and contribute to ferroptosis, a form of cell death characterized by the accumulation of lethal lipid peroxidation products [48]. Depletion of glutathione peroxidase 4 (GPX4), a key lipid detoxification enzyme, exacerbates this condition, increasing a cell’s susceptibility to ferroptosis [48].

In our studies on cancer cell membrane fluidity, we focused on both the outer (hydrophilic) and deeper (hydrophobic) regions. While we observed no statistically significant changes in the hydrophilic lipid heads, the tested compounds caused significant membrane stiffening in the hydrophobic regions of unsaturated fatty acid chains. This effect is likely related to ROS action and lipid peroxidation. We supported this hypothesis by demonstrating that pre-incubation with the antioxidant NAC significantly reduced lipid peroxidation. A decrease in membrane fluidity has serious consequences for cancer cells, leading to increased permeability, impaired membrane protein function, and altered enzyme activity.

Studies on the structural properties of flavonoids confirm their ability to influence cell membrane fluidity. Günther et al. [51] found that the localization and reactivity of flavonoids, such as kaempferol and morin, in lipid environments directly correlate with changes in membrane fluidity [51]. Furthermore, some studies highlight the modulatory effect of flavonoid derivatives. Ulrih et al. [52] reviewed the effects of specific polyphenols, noting that many of them tend to reduce cell membrane fluidity, likely through oxidative modifications that can alter protein functionality and cell signaling. Additionally, Dudek et al. [53] showed that halogenated flavonoids can interact with lipid bilayers, affecting their fluidity and exerting a cytotoxic effect on cancer cells while sparing healthy cells. This highlights their therapeutic potential in modulating membrane properties in malignant tumors [53]. Undeniably, understanding the dynamics of lipid peroxidation in cancer cells can lead to innovative therapeutic approaches that enhance the efficacy of existing treatments. Strategies targeting ROS generation can induce lipid peroxidation, thereby sensitizing tumors to ferroptosis and increasing the effectiveness of chemotherapy and immunotherapy [54]. The lipid peroxidation observed in this study, along with the resulting increase in membrane stiffness, should not be interpreted solely as a passive byproduct of oxidative stress but rather as an active factor promoting apoptosis. According to the literature, changes in the physicochemical properties of membranes induced by ROS constitute a critical link mediating the transition from early chemical stress to activation of the enzymatic cascade [55].

The mechanism of lipid peroxidation begins with the reaction of ROS with polyunsaturated fatty acids (PUFAs) in membranes, leading to the formation of lipid radicals and secondary peroxidation products such as malondialdehyde (MDA) and 4-hydroxy-2-nonenal (4-HNE)—compounds that are both cytotoxic and capable of modifying protein function and membrane integrity [56]. Studies have shown that excessive lipid peroxidation can alter the physicochemical properties of membranes, including reduced fluidity, depolarization, and disrupted lipid asymmetry, which may favor membrane permeabilization and organelle dysfunction, including mitochondrial impairment [57]. Lipid peroxidation may function both as an initiating element of a signaling cascade—lowering membrane barriers, facilitating mitochondrial dysfunction, and promoting cytochrome c release—and as a secondary marker of oxidative damage that accumulates in response to other forms of oxidative stress [56]. In regulated cell death, such as ferroptosis, lipid peroxidation plays a key executioner role and is a primary driver of cell death; in classical apoptosis, its role is less dominant and more ancillary, highlighting the complex interplay between ROS, membrane alterations, and activation of executioner enzymes [58].

The proposed sequence of events suggests that rapid ROS accumulation leads to oxidation of PUFAs, directly resulting in the reorganization of membrane microdomains and a decrease in their fluidity. This phenomenon is fundamental for mitochondrial function; loss of optimal mitochondrial membrane fluidity promotes pore formation (Mitochondrial Permeability Transition Pore, MPTP) and cytochrome c release [59]. Our results, indicating a significant increase in DAUDA probe polarization in the hydrophobic region, suggest that these profound structural membrane changes precede the activation of caspase-9 and caspase-3. Moreover, modifications of the lipid environment can directly affect the conformation and activity of membrane-associated signaling proteins, such as MAPK family kinases, further amplifying the pro-apoptotic signal [60].

Thus, changes in membrane fluidity represent an integral component of the “point of no return” in programmed cell death, integrating primary oxidative damage with the execution phase of apoptosis. Accordingly, the observations reported here regarding lipid peroxidation and membrane stiffening, while consistent with existing mechanistic concepts, need not in themselves constitute the sole cause of caspase activation and apoptosis; they may also reflect accumulating oxidative stress that integrates multiple signals leading to cell death. In light of these findings, we propose a model in which membrane peroxidation and associated structural changes reinforce the signals driving apoptosis.

## 4. Proapoptotic Activity of Flavanone/Chromanone Derivatives—Caspase-3 and Caspase-9 Activation

To confirm that the evaluated derivatives induce programmed cell death (PCD) in colon cancer cells, we analyzed the activity of caspase-3 and -9. These caspases are integral to the mitochondrial (intrinsic) pathway of apoptosis. Caspase-9 acts as an initiator caspase for PCD, while caspase-3 is the primary executioner (effector) enzyme, responsible for the final stages of apoptosis and complete cellular disintegration [61].

**Figure 4:**
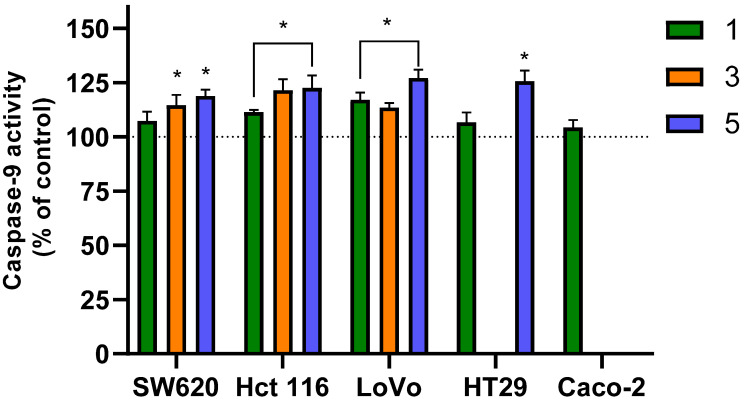
Measurement of caspase-9 activity in HCT 116, SW620, LoVo, Caco-2 and HT-29 cells exposed to compound **1**, **3** and **5**. Cells were incubated with derivatives for 24 h at concentrations of the calculated IC_50_. The results represent mean ± SD of the data from two individual experiments, untreated control cells arbitrarily taken as 100%, * *p* < 0.05 vs. control.

**Figure 5:**
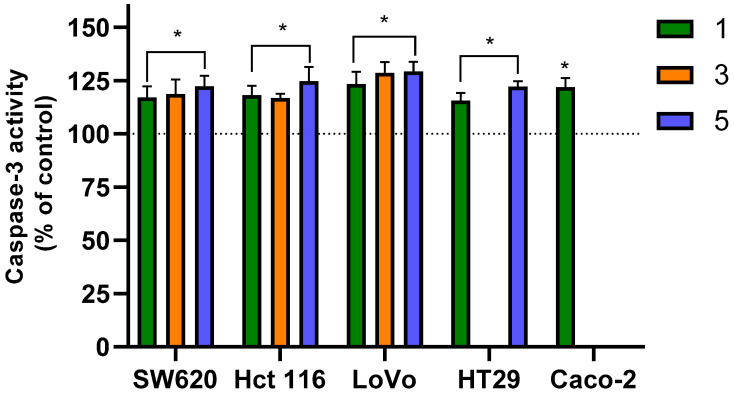
Measurement of caspase-3 activity in HCT 116, SW620, LoVo, Caco-2 and HT-29 cells exposed to compound **1**, **3** and **5**. Cells were incubated with derivatives for 24 h at concentrations of the calculated IC_50_. The results represent mean ± SD of the data from two individual experiments, untreated control cells arbitrarily taken as 100%, * *p* < 0.05 vs. control.

After a 24 h incubation of cancer cells with the tested derivatives, we observed a clear increase in the activity of both caspase-9 and -3 (Figure 4 and Figure 5, respectively), which confirms the activation of apoptotic pathways. For the initiator caspase-9, a statistically significant increase in activity was noted in the majority of experimental variants, ranging from approximately 15% to 25% depending on the compound and the cell line. The only exceptions were with derivative **1**, which showed no increase in caspase-9 activity in SW620, HT29, and Caco-2 cells. Among the three derivatives, compound **5** was the most effective at inducing caspase-9 activation across all cell lines.

The flavanone/chromanone derivatives also caused a significant increase in the activity of the active form of caspase-3 (caspase-3 cleaved at Asp175/Ser176). Unlike caspase-9, statistically significant increases in this effector enzyme’s activity were observed for all tested analogues and cell lines. It is also important to note that the increase in caspase-3 activity was approximately 5–10% higher than that of caspase-9 in each experimental variant. The largest increase in caspase-3 activity was observed in the LoVo line, where it was approximately 25–30% compared to control cells for all three derivatives. For the remaining cell lines, activation of effector caspase-3 was only slightly lower, ranging from ~15–20%.

In the initial phase of our studies on flavanone/chromanone derivatives, we focused on measuring changes in mitochondrial membrane potential. We observed strong hyperpolarization and local depolarization, which are characteristic steps in the apoptotic process. This indicated that the derivatives were likely activating programmed cell death (PCD) in colon cancer cells [28]. Our analysis of caspase-3 and -9 activity confirmed these proapoptotic properties. The increased activity of these cysteine proteases is associated with the activation of the intrinsic (mitochondrial) apoptotic pathway. The signaling pathways mediating these effects often converge on mitochondrial dysfunction, which leads to the release of proapoptotic factors and the subsequent activation of caspases [62]. Our findings correspond to the results of from other research groups. Numerous publications confirm the effective proapoptotic properties of flavonoid derivatives against colon cancer cells, including their ability to activate caspases [63,64,65]. A focused study by Palko-Łabuz et al. [66] investigated the proapoptotic mechanisms of specific flavanone derivatives and found an increase in caspase-3 activity, a key factor in apoptosis [66]. Importantly, several studies point to a significant contribution of reactive oxygen species (ROS) generated by flavonoid derivatives in inducing cancer cell apoptosis [63,65]. For example, Choi et al. [63] reported that kaempferol led to increased activation of caspase-3, -8, and -9 through ROS production in HCT116 and HCT15 colon cancer cells, demonstrating the role of oxidative stress in initiating apoptotic pathways [63]. Similarly, Wongkaewkhiaw et al. [65] found that pinostrobin, a flavanone from lemongrass, exhibits significant antiproliferative activity and induces apoptosis in cancer stem cells via a ROS-dependent mechanism [65]. Our results correspond with these reports, especially considering the role of ROS in the overall cytotoxic capacity of the derivatives. In the first part of our study, we showed that inhibiting the prooxidant activity of derivatives **1**–**5** with antioxidants (NAC or Trolox) significantly reduced their cytotoxicity [28]. This conclusion was further supported by our analysis of changes in cell membrane fluidity and lipid peroxidation. Therefore, modulating oxidative stress appears to be an important mechanism by which flavanones exert their anticancer effects, highlighting the link between the dual antioxidant/prooxidant nature of these molecules and proapoptotic signaling [67].

The interactions between these apoptotic signaling pathways and flavonoid derivatives may define new therapeutic strategies for colon cancer. The activation of initiator caspases, primarily caspase-9, leads to the further activation of effector caspases like caspase-3, highlighting their crucial role in apoptosis. The enhanced ROS production and subsequent signaling through caspase activation demonstrate a potent mechanism by which these compounds can trigger apoptotic events in cancer cells.

## 5. Effects of Flavanone/Chromanone Derivatives on MAPK

**Figure 6:**
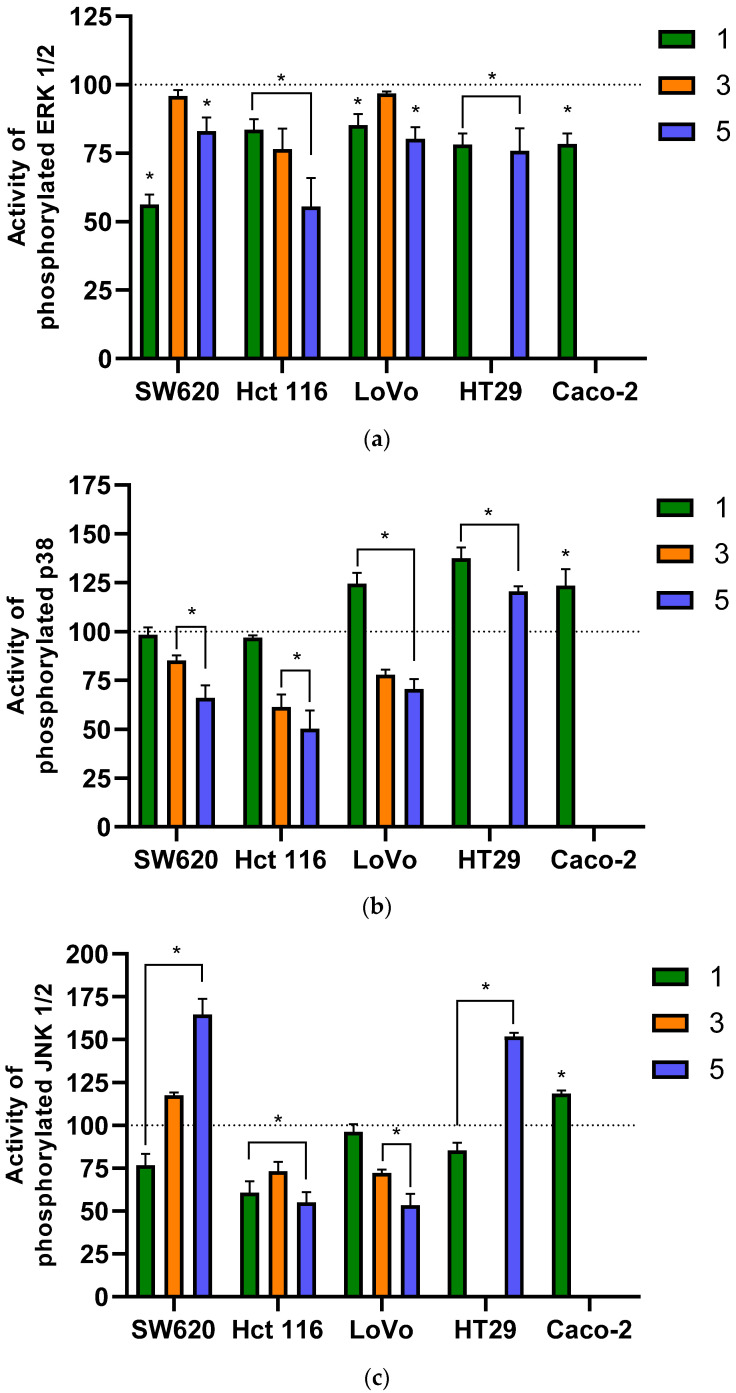
(**a**) Measurement of phosphorylated kinase ERK 1/2 activity in HCT 116, SW620, LoVo, Caco-2 and HT-29 cells exposed to compound **1**, **3** and **5**. Cells were incubated with derivatives for 24 h at concentrations of the calculated IC_50_. The results represent mean ± SD of the data from 2 individual experiments, untreated control cells arbitrarily taken as 100%, * *p* < 0.05 vs. control. (**b**) Measurement of phosphorylated kinase p38 activity in HCT 116, SW620, LoVo, Caco-2 and HT-29 cells exposed to compound **1**, **3** and **5**. Cells were incubated with derivatives for 24 h at concentrations of the calculated IC_50_. The results represent mean ± SD of the data from two individual experiments, untreated control cells arbitrarily taken as 100%, * *p* < 0.05 vs. control. (**c**) Measurement of phosphorylated kinase p38 activity in HCT 116, SW620, LoVo, Caco-2 and HT-29 cells exposed to compound **1**, **3** and **5.** Cells were incubated with derivatives for 24 h at concentrations of the calculated IC_50_. The results represent mean ± SD of the data from two individual experiments, untreated control cells arbitrarily taken as 100%, * *p* < 0.05 vs. control.

Mitogen-activated protein kinases (MAPK) are central to numerous cellular processes. This pathway transmits biochemical signals from the cell surface to the nucleus, where it activates transcription factors involved in critical functions such as cell division, growth, migration, differentiation, and apoptosis. Abnormal regulation of MAPK activity, often caused by genetic mutations, is a common feature of cancer. Such dysregulation can lead to uncontrolled proliferation, migration, and invasion, along with a lack of apoptosis. Because of the MAPK pathway’s key role in carcinogenesis, kinases such as RAF, MEK, and ERK are important targets for modern cancer therapies [68,69]. We used a fluorometric ELISA kit to determine the concentration of the active (phosphorylated) forms of ERK 1/2, JNK, and p38 kinases. Changes in the activity of individual kinases are shown in Figure 6a–c.

After a 24 h exposure of colon cancer cells to the tested derivatives, we observed a clear and statistically significant decrease in the concentration of phosphorylated ERK 1/2 in most experimental variants. Derivative **3** had the weakest effect in this regard, causing a statistically significant reduction in ERK 1/2 concentration (approximately 25% compared to the control) only in HCT116 cells. No changes were observed in the SW620 and LoVo lines. In contrast, exposure to derivatives **1** and **5** resulted in a clear decrease in phosphorylated ERK 1/2 concentration, ranging from approximately 15% to 25% relative to the untreated control. Two results are particularly noteworthy: compound **1** in SW620 cells and compound **5** in the HCT116 cell line. In both cases, a very strong decrease in phosphorylated ERK 1/2 concentration of up to ~45% was observed, indicating potent kinase inhibition.

The kinetics of changes in the concentration of active p38 and JNK kinases differed entirely from those of ERK 1/2. In the case of p38 kinase, both increases and decreases in phosphorylated p38 concentration were observed, depending on the cell line. For the HT29 and Caco-2 lines, incubation with derivatives **1** and **5** resulted in a significant increase in p38 concentration (approximately 25–35% compared to the control). A similar increase of approximately 25% was also seen after incubating LoVo cells with analogue **1**. However, the compound **1** was less active against the SW620 and HCT116 lines, where no statistically significant changes were observed. Conversely, incubation of SW620, HCT116, and LoVo cells with derivatives **3** and **5** led to a significant decrease in phosphorylated p38 concentration, reaching up to 50% in the most effective case (derivative **5** in the HCT116 line). The mean percentage decrease in p38 concentration ranged from ~15% to 35%.

We observed a similar diversity in concentration changes for the JNK kinase across different cancer cell lines. The tested derivatives caused both an increase and a decrease in phosphorylated JNK concentration, depending on the specific compound and cell line. Notably, each derivative contributed to both a decrease and an increase in JNK concentration in different colon cancer cell lines.

For derivative **1**, a decrease in JNK protein concentration was observed in four of the five cell lines used. Only in the case of Caco-2 cells did incubation with compound **1** cause a ~20% increase in JNK concentration. For the other four cell lines, a statistically significant decrease of approximately 15–35% was observed relative to the control, with the exception of the LoVo line, which showed no statistically significant changes. Derivative **3** caused a ~15% increase in JNK levels in the SW620 line, while a similar decrease of approximately 15–20% was observed in the HCT116 and LoVo lines compared to control cells. The last derivative, derivative **5**, showed a similar trend in the kinetics of JNK level changes. Interestingly, this compound, as with the other two kinases (ERK 1/2 and p38) also caused the largest concentration changes for JNK. For derivative **5**, a very significant increase in phosphorylated JNK concentration was observed in the SW620 and HT29 lines, reaching approximately 35–50% compared to the control. Conversely, incubating HCT116 and LoVo cells with derivative **5** led to a decisive decrease in JNK levels of up to ~45%.

These results highlight the very complex mechanism of biological activity of the analyzed flavanone/chromanone derivatives and their underlying anticancer properties.

The mitogen-activated protein kinase (MAPK) pathway is a central regulator of tumorigenesis and cancer progression. This complex signaling cascade controls fundamental cellular processes, including proliferation, differentiation, apoptosis (cell death), and migration. In cancer, the MAPK pathway—particularly the RAS/RAF/MEK/ERK subfamily—is frequently characterized by abnormal or excessive activation due to genetic mutations or epigenetic alterations. This uncontrolled signaling drives hallmarks of cancer, such as unrestricted cell proliferation, inhibited apoptosis, angiogenesis, metastasis, and chemoresistance [70]. The highly complex nature of the MAPK pathway in cancer is evident in the fact that its components cannot be unequivocally defined as solely tumor promoters or suppressors; their activity levels vary depending on the tumor type and disease stage. While ERK is typically a pro-oncogenic kinase with increased activity, JNK and p38 exhibit more diverse roles. They can act as factors promoting cell death (suppressors) or as promoters of cancer cell invasion and proliferation. The key to understanding pathogenesis often lies in analyzing the ratio of activity between these three pathways within a given tumor type [68].

Our research supports these conclusions. In the case of ERK kinase, we observed a consistent decrease in the activity of its phosphorylated form across nearly all experimental variants, regardless of the cell line or derivative used. However, the change in activity was not as clear-cut for the other two kinases, p38 and JNK1/2, where we observed either an increase or a decrease in their concentration, depending on the specific cell line.

The ability of flavonoid compounds to modulate the MAPK signaling cascade is supported by numerous scientific studies [68]. The recent work by Balkrishna et al. [71], provided crucial information in this regard, confirming through computational analyses the capacity of flavanones to inhibit proteins involved in the MAPK pathway. Their results indicate a significant relationship between the structural features of flavanones and their effectiveness in disrupting key signaling pathways in malignant tumors [71].

The observed reduction in ERK 1/2 activity correlates with findings from other research groups, underscoring the potential of flavanone derivatives as ERK inhibitors. For instance, curarione, a lavandulyl flavanone, has been shown to significantly attenuate ERK signaling and consequently reduce cell proliferation in cancer models. This mechanism involves the inhibition of RSK2 and subsequent NF-κB signaling, both processes downstream of ERK activation [72]. Similarly, studies by Liu et al. [73] indicate that berberine inhibits the EGFR-RAF-MEK-ERK signaling pathway in glioma cells, leading to cell senescence and reduced proliferation [73]. Furthermore, several reports indicate that the attenuation of ERK 1/2 signaling results in reduced metastasis and invasiveness of breast cancer cells (MCF-7) and ultimately leads to their death via apoptosis or autophagy [74,75,76,77]. Flavonoids are also known to modulate key cellular processes by affecting the phosphorylation of proteins involved in the ERK pathway. Baek et al. [78] demonstrated that eriocitrin can inhibit angiogenesis by disrupting VEGFR2-dependent signaling pathways, which are closely linked to ERK signaling. This suggests a synergistic relationship in which flavanones may not only directly inhibit ERK but also influence upstream signaling cascades [78].

Our studies on JNK and p38 kinase activity suggest a dual and complex role for these proteins in cancer progression, even within cells of the same tumor type. As our results indicate, the increase or decrease in JNK and p38 kinase activity correlated precisely with specific cell lines. Notably, the same compound, such as derivative **5**, was capable of both activating and inhibiting the same kinase depending on the cellular context.

This complexity in the biological activity of JNK and p38 in the context of anticancer properties is also highlighted in studies by other teams using flavonoids. It appears that, depending on the type and stage of cancer, flavonoid derivatives can, similar to our observations, cause both increased and inhibited JNK and p38 activity. It is important to emphasize that in both modulation variants (activation and inhibition), the result was often cancer cell apoptosis, decreased adhesion, migration, invasion, and cell cycle inhibition [79,80,81,82,83]. In light of our results, it is crucial to recognize the multifaceted characteristics of JNK and p38. In response to severe cellular stress, JNK can act as a tumor suppressor by inducing apoptosis. However, in many types of cancer (especially in advanced stages), increased JNK activity is observed, promoting cell survival, invasion, and metastasis [82]. Similarly, while numerous studies provide experimental evidence for the anti-cancer role of p38, many others show that this kinase promotes cancer development by increasing survival, migration, or resistance to stress and chemotherapeutic agents in cancer cells [84].

JNK and p38 kinases are classically activated in response to stress, yet their impact on apoptosis can be either pro- or anti-apoptotic depending on the intensity and duration of the signal as well as the cell type. For example, studies in both normal and cancer cell models have shown that JNK can mediate apoptosis by promoting the translocation of pro-apoptotic proteins Bax/Bak and mitochondrial destabilization, while short-term or moderate JNK activation may participate in adaptive mechanisms supporting cell survival under oxidative stress [85]. Similarly, p38 MAPK can stimulate apoptotic processes through phosphorylation and activation of pro-apoptotic factors, while also regulating cellular responses to ROS; however, its role may be modulated by crosstalk with other signaling pathways [86].

Importantly, the variability in JNK/p38 responses observed in our study depending on the cell line is characteristic of colorectal cancer and often reflects differences in the mutational status of *KRAS* and *TP53* genes, which determine the baseline sensitivity of cells to stress stimuli [87,88]. Therefore, the differential JNK/p38 modulation observed in our models may reflect variations in the redox state of individual cell lines, differing thresholds of sensitivity to oxidative stress, and distinct adaptive programs. In some lines, strong ROS generation may lead to JNK/p38 activation as part of a direct pro-apoptotic signal, as has been proposed for certain chemotherapeutics and pro-oxidant compounds [89]. In other lines, the response may manifest as inhibition of adaptive or compensatory signaling, resulting in divergent kinase phosphorylation profiles and corresponding biological effects on cell survival.

Such heterogeneity has also been described in H_2_O_2_-induced models, where JNK and p38 activation was necessary to induce apoptosis, whereas inhibition of these kinases increased cell survival under oxidative stress conditions [90]. Moreover, evidence indicates that interactions between ROS and MAPKs are bidirectional: ROS can activate JNK/p38, but activation of these kinases can in turn influence ROS generation and amplify oxidative stress via feedback mechanisms and inhibition of MAPK phosphatases, leading to prolonged kinase activation [91]. This feedback mechanism may contribute to inter-line differences depending on the expression of phosphatases, redox-sensitive regulators, and other modulatory factors affecting signaling.

For these reasons, we propose that the diverse effects of JNK and p38 modulation observed in our cell lines reflect the biological heterogeneity of stress signaling in colorectal cancer cells and their differing thresholds of antioxidant capacity. In this model, ROS act as key mediators integrating stress signals and activating the appropriate MAPK pathways.

## 6. Analysis of Changes in Nrf2 Concentration

Nuclear factor erythroid 2-related factor 2 (Nrf2) is a key transcription factor that regulates the expression of genes involved in redox processes, xenobiotic metabolism, DNA repair, and protein and lipid homeostasis. Due to its central role in controlling oxidative stress, Nrf2 is a significant target for therapies aimed at oxidative stress-related diseases, including cancer [92]. Overexpression of Nrf2 in cancer cells can protect them from the cytotoxic effects of treatment and is associated with tumor progression and chemoresistance. Therefore, the Nrf2-antioxidant response element (ARE) pathway is considered an ideal target for chemopreventive agents [93,94].

**Figure 7:**
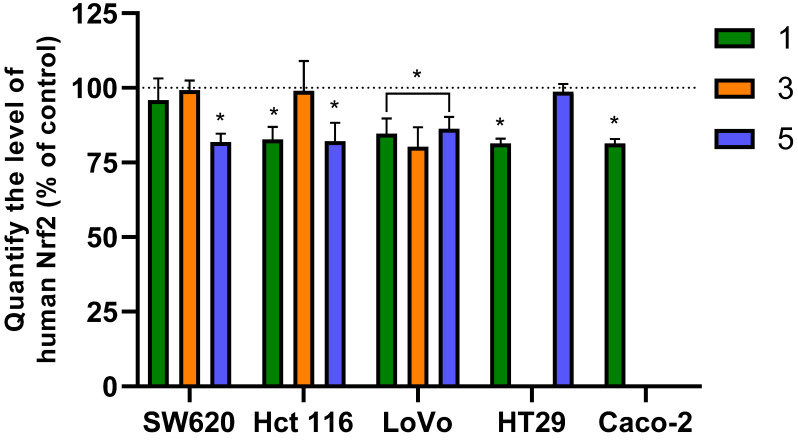
Measurement of Nrf2 activity in HCT 116, SW620, LoVo, Caco-2 and HT-29 cells exposed to compound **1**, **3** and **5**. Cells were incubated with derivatives for 24 h at concentrations of the calculated IC_50_. The results represent mean ± SD of the data from 2 individual experiments, untreated control cells arbitrarily taken as 100%, * *p* < 0.05 vs. control.

Our analysis of the Nrf2 transcription factor in colon cancer cells showed that the three flavanone/chromanone derivatives were capable of reducing its levels after a 24 h incubation (Figure 7). The decrease in Nrf2 concentration correlated with the specific derivative used. Compound 3 had a significantly weaker effect; it caused a statistically significant decrease in Nrf2 levels (approximately 20% compared to the control) only in LoVo cells. In contrast, no statistically significant differences in Nrf2 levels were observed in SW620 cells treated with derivative 1 or in the HT29 cell line exposed to compound **5**. However, in the remaining experimental variants, a significant decrease in Nrf2 concentration was observed, ranging from 15 to 20% compared to untreated control cells. These results align with our previous findings regarding the prooxidant properties of the analyzed derivatives [28].

The Nrf2 transcription factor is a crucial regulator of cellular defense, playing a fundamental role in maintaining redox homeostasis (oxidation-reduction balance) within the body. It safeguards cells from damage by activating the expression of over 250 target genes, which encode cytoprotective proteins, antioxidant enzymes, and xenobiotic-metabolizing enzymes [94]. Nrf2 overactivity is frequently observed across many cancers, often resulting from mutations in its inhibitor, the *KEAP1* gene, or in the *Nrf2* gene itself (NFE2L2). In colorectal cancer (CRC), the Nrf2 pathway is a key mediator of cellular defense against oxidative stress and inflammation, which are known to initiate CRC progression and contribute to therapeutic resistance. Studies consistently show that elevated Nrf2 signaling in CRC cells significantly promotes tumor growth and chemoresistance in various models [95,96,97,98]. For instance, O’Cathail et al. [95] highlighted that aberrant Nrf2 signaling may mediate resistance to chemoradiotherapy in CRC through metabolic reprogramming.

Our research confirms that the Nrf2 transcription factor is one of the molecular targets of the anticancer activity exerted by the studied flavonoid derivatives. The observed decrease in Nrf2 activity in colon cancer cells following exposure to the compounds is indicative of their broad spectrum of biological activity. Importantly, this reduced Nrf2 activity in cancer cells is linked to the prooxidant properties of the compounds and their resulting anticancer effects. Our findings suggest that, in addition to the direct generation of ROS in cancer cells, the severe oxidative stress induced across various colon cancer cell types is also associated with a reduction in Nrf2 and glutathione levels.

Our results are strongly supported by numerous studies from other research teams, demonstrating that targeting Nrf2 with flavonoid compounds is a promising strategy in modern anticancer therapies. Multiple publications indicate that natural flavonoids, such as naringin [99] or luteolin [100], can act not only as protective agents against oxidative damage but also as factors that disrupt Nrf2-dependent survival pathways in CRC. Furthermore, flavonoid compounds have been shown to inhibit Nrf2 activity in HT29 cells while simultaneously increasing ROS, which strongly correlates with our findings [101,102,103]. Disturbances in free radical metabolism led to increased sensitivity of cancer cells to chemotherapy, consequently enhancing apoptotic cell death [101,102,103]. Chikkegowda et al. [104] emphasized that the design and synthesis of molecular modulators targeting Nrf2 pathways show promise in limiting CRC cell proliferation. They showed that exposing cancer cells to one such compound reduced tumor growth in Nrf2-expressing colorectal cancer models, confirming the therapeutic potential of appropriately selected Nrf2 modulators [104]. In a related approach, Ren et al. [105] used brusatol to emphasize the significant therapeutic benefits of a multifaceted strategy targeting the Nrf2 axis. The use of flavanone derivatives, both as standalone agents and in combination with conventional therapies, may not only inhibit tumor growth but also increase the efficacy of existing chemotherapy regimens, particularly in patients with Nrf2 pathway dysregulation [83].

A key issue in interpreting the mechanism of action of the studied flavanone derivatives is establishing the hierarchy of molecular events, particularly the role of reactive oxygen species (ROS) as the primary trigger of the apoptotic cascade. While our study emphasizes the central role of oxidative stress in the cytotoxic effects of the compounds under investigation, it should be noted that the role of ROS as an initiating event versus a secondary consequence of broader cellular stress has not yet been fully resolved in the literature [106]. On the other hand, an emerging body of research indicates that oxidative stress products can act both as a cause and as a consequence of the apoptotic process. In many models, ROS are generated rapidly following stimulation, suggesting their role as an early stress signal capable of activating ASK1, MAPKs (including JNK and p38), and other regulatory proteins responsible for determining the cell’s entry into apoptotic death [40]. However, in other contexts, ROS are generated secondarily, for instance through damage to the mitochondrial respiratory chain or as a result of the release of pro-apoptotic mitochondrial proteins, which may further amplify oxidative stress and constitute a feedback component of the cell death mechanism Chandimali [107]. Such bidirectional relationships indicate that the sequence of events—ROS accumulation, membrane lipid peroxidation, loss of mitochondrial integrity, and caspase activation—can be dynamic and dependent on the type of stimulus, cell type, and cellular redox state. The literature suggests that ROS may function both as initiating stress signals and as amplifiers of the apoptotic response, particularly when the cell’s antioxidant capacity is overwhelmed [56].

Although oxidative stress is sometimes interpreted in the literature as a secondary phenomenon following progressive cellular degradation, our results suggest that, in the case of the tested compounds 1, 3, and 5, ROS generation constitutes an initiating event. This hypothesis is strongly supported by experiments employing N-acetylcysteine (NAC)—preincubation with this antioxidant almost completely abolished the cytotoxicity of the compounds and prevented changes in cell membrane fluidity, demonstrating that, in the absence of an initial oxidative impulse, subsequent stages of damage do not occur. According to the proposed model, the sequence of events begins with a rapid accumulation of ROS, leading to peroxidation of membrane lipids. This phenomenon, observed in our study as a decrease in fluidity in the hydrophobic core of the bilayer (reduced DAUDA probe polarization), induces structural changes in organelle membranes, particularly in mitochondria. Disruption of mitochondrial membrane integrity and loss of membrane potential (ΔΨm) are direct consequences of peroxidation, enabling the release of cytochrome c into the cytosol. This chronology—from oxidative stress through physicochemical membrane damage to caspase activation—is consistent with the classical model of mitochondria-mediated apoptosis induced by xenobiotics [106,108].

The scientific literature emphasizes that ROS can act as ‘second messengers,’ which, through modulation of MAPK pathways (demonstrated in this study by ERK1/2 inhibition and p38/JNK activation) and inhibition of the Nrf2 system, permanently shift cellular equilibrium toward apoptosis [109]. Therefore, the observed activation of initiator caspase-9 and effector caspase-3 does not appear to be a nonspecific consequence of cell disintegration, but rather the final step of a precisely defined signaling pathway in which oxidative stress plays a predominant and initiating role. Our results are consistent with a model in which oxidative stress functions as a critical mediator and potential initiating signal of the cascade leading to apoptosis, although we recognize that the exact molecular chronology requires further investigation, ideally using methods capable of detecting ROS and membrane changes at very early time points in real time.

Given the compelling evidence linking Nrf2 to colorectal carcinogenesis and treatment resistance, combined with the encouraging results from studies on flavanone derivatives, continuing this line of research is well-justified.

## 7. Conclusions and Future Perspectives

This article continues the research on flavanone/chromanone derivatives, complementing and substantially expanding upon previously presented results [20]. The current study examines the multifaceted anticancer activity of these novel derivatives in colon cancer cell lines. The main focus was placed on elucidating the mechanisms related to apoptosis induction, the generation and role of oxidative stress, and the involvement of key signaling pathways in cell proliferation.

Our results clearly demonstrate that these derivatives possess potent proapoptotic properties driven by a complex interplay of cellular events. The primary mechanism involves the induction of significant oxidative stress, which is supported by the observed lipid peroxidation and subsequent stiffening of cell membranes, predominantly in the hydrophobic inner layers. The crucial role of ROS in the overall anticancer activity was confirmed by showing that preincubation with the antioxidant NAC significantly reduced both lipid peroxidation and cytotoxicity. This oxidative attack directly leads to the activation of the intrinsic apoptotic pathway, as manifested by the marked cleavage and degradation of the PARP enzyme–a highly reliable marker of cell death—and subsequent activation of initiator caspase-9 and executioner caspase-3. Furthermore, the tested derivatives actively disrupt key signaling pathways that promote cancer cell survival and proliferation. They consistently inhibit the pro-oncogenic ERK 1/2 MAPK pathway in most cell lines, while simultaneously exerting dual, cell-line-dependent modulation of JNK and p38 kinases. Crucially, these compounds effectively inhibit the Nrf2 transcription factor, a key regulator of cellular defense and a mediator of chemoresistance in colorectal cancer.

The overall conclusion is that these flavanone/chromanone derivatives act as potent anticancer agents by simultaneously eliminating cellular antioxidant mechanisms through ROS generation and blocking multiple survival and proliferation signals (ERK 1/2, Nrf2), resulting in effective programmed cell death.

It should be emphasized that the results presented in this study were obtained exclusively in in vitro models, which entails well-known limitations regarding the direct extrapolation of these observations to in vivo conditions. In particular, cellular systems do not fully recapitulate the complex interactions present in the tumor microenvironment, such as the influence of the extracellular matrix, stromal cells, the immune system, or variable metabolic conditions. Furthermore, the present study did not include an evaluation of drug development-relevant properties, such as the aqueous solubility, metabolic stability, or pharmacokinetics of the compounds investigated. Although the flavanone scaffold is generally regarded as a drug-like chemical framework, variations in lipophilicity and substitution patterns may significantly influence bioavailability, tissue distribution, and in vivo clearance. Consequently, the lack of pharmacokinetic data limits direct conclusions regarding the therapeutic potential of the analyzed derivatives beyond cell-based systems.

For these reasons, it is essential to clearly distinguish between direct experimental observations and the proposed mechanistic interpretations, which should be considered as model-based hypotheses requiring further validation. We have directly demonstrated that the studied derivatives induce ROS accumulation, alterations in cellular membrane properties, modulation of selected MAPK signaling pathways, and caspase activation leading to apoptosis in colorectal cancer cells. The proposed mechanistic model, integrating oxidative stress, membrane structural perturbations, and MAPK signaling regulation as a coordinated sequence of events culminating in cell death, represents an interpretation based on correlations and consistency with the literature rather than definitive evidence of causal relationships.

Specifically, while data obtained using antioxidants and the observed sequence of changes are consistent with a hypothesis in which oxidative stress functions as a key mediator of the apoptotic response, they do not allow for a definitive conclusion as to whether ROS constitute an initiating event or rather a secondary component within the broader landscape of cellular stress. Similarly, changes in membrane fluidity and modulation of MAPK activity may contribute to apoptosis activation but may also reflect adaptive or compensatory cellular responses to accumulating oxidative damage.

Therefore, the presented findings should be considered hypothesis-generating and provide a rationale for future studies focused on high temporal-resolution kinetic analyses, selective modulation of individual signaling pathways, physicochemical characterization, metabolic profiling, and in vivo validation in appropriate colorectal cancer models to assess both the mechanistic and therapeutic relevance of the analyzed derivatives.

Future plans therefore require preclinical studies in animal models to assess systemic toxicity, bioavailability, and therapeutic efficacy. Furthermore, further studies should be dedicated to detailed molecular investigations, such as structure–activity relationship (SAR) analysis and specific binding assays, to precisely define the molecular targets of the most active compounds (derivatives **1**, **3**, and **5**) and investigate their potential as synergistic agents in combination with existing chemotherapies in patients with Nrf2 dysregulation or other mutations in DNA repair pathways.

## 8. Materials and Methods

### 8.1. Biological Material, Culture and Passage of Cells

In vitro assays were carried out to assess the biological effects of the flavanone/chromanone derivatives against a broad spectrum of human cancer cell lines. The selection of these cell lines was based on their molecular and genetic heterogeneity. The study incorporated a total of 5 human adherent colon cancer lines: HCT 116 (ATCC^®^ CCL-247^TM^; colorectal cancer, Dukes’ type A), SW620 (ATCC^®^ CCL-227^TM^; colorectal adenocancer, Dukes’ type C), LoVo (ATCC^®^ CCL-229^TM^, colorectal adenocancer, Dukes’ type C, grade IV), Caco-2 (ATCC^®^ HTB-37^TM^, colorectal adenocancer, Dukes’ type B) and HT-29 (ATCC^®^ HTB-38^TM^, colorectal adenocancer, Dukes’ type C). All these cell lines were purchased from American Culture Collection (ATCC), Manassas, VA, USA.

The specific culture media for each cell line were prepared to support optimal growth. HCT 116, SW620 and LoVo cancer cells lines were cultured in Dulbecco’s minimal essential medium (DMEM, Lonza, Visp, Switzerland) culture medium supplemented with 10% fetal bovine serum and antibiotics (10 U/mL penicillin and 0.5 mg/mL streptomycin). Caco-2 cell line was culture in Eagle’s Minimum Essential Medium (EMEM, Sigma-Aldrich; St. Louis, MO, USA) culture medium supplemented with 20% fetal bovine serum and antibiotics (10 U/mL penicillin and 0.5 mg/mL streptomycin). HT-29 tumor cells were cultured in RPMI (Thermo Fisher Scientific, Waltham, MA, USA) culture medium supplemented with 10% fetal bovine serum and antibiotics (10 U/mL penicillin and 0.5 mg/mL streptomycin).

All cell cultures were maintained in sterile, single-use vessels under controlled conditions within a standard CO_2_ incubator (37 °C, 5% CO_2_, 95% air, 100% relative humidity). To ensure the cells remained in the exponential growth phase, routine subculturing was performed 2–3 times per week, typically when the cell monolayer reached approximately 80% confluence.

The procedure for subculturing involved several steps. First, the existing culture medium was aspirated, and the cell monolayer was washed with a 0.9% NaCl solution. A 0.25% trypsin-EDTA solution (Gibco, Grand Island, New York, NY, USA) was then added to the vessel, in a volume appropriate for the surface area (300–500 μL). The cells were incubated with the trypsin solution for 3–5 min inside the CO_2_ incubator, with the detachment process monitored via microscopy. Once the cells had dislodged, fresh culture medium was added to the vessel to inactivate the trypsin. The resulting cell suspension was homogenized and then transferred to new sterile vessels at a density suitable for subsequent experiments.

### 8.2. Measurement of Membrane Fluidity

Membrane fluidity and any localized changes within the cell membrane were evaluated using the fluorescence anisotropy of two distinct probes: 1-(4-(trimethylamino) phenyl)-6-phenylhexa-1,3,5-triene (TMA-DPH) and 11-(Dansylamino)undecanoic acid (DAUDA) (Sigma-Aldrich; St. Louis, MO, USA). DAUDA was utilized to assess the hydrophobic core of the lipid bilayer, while TMA-DPH was positioned to analyze the more hydrophilic region near the membrane surface. An increase in fluorescence anisotropy indicates a corresponding decrease in membrane fluidity. A final concentration of 1 μM for each fluorescent probe was used in the experimental solutions.

Prior to the experiment, cells were seeded in 6-well plates and incubated for 24 h. Following this initial incubation, the compounds under investigation were introduced to the cell cultures at their predetermined IC_50_ concentrations. In the antioxidant variant, cells were preincubated for 1 h with N-acetylcysteine (NAC) (Sigma-Aldrich; St. Louis, MO, USA). After this time, the antioxidants were washed away, and the tested derivatives were added to the cells at IC_50_ concentrations.

After an additional 24 h incubation period with the compounds, the cells were detached using trypsin and transferred to Eppendorf tubes. The resulting cell pellets were washed twice with cold (0–4 °C) phosphate-buffered saline (PBS). After removing the PBS, a cold (0–4 °C) Tris-HCl/KCl buffer (50 mM Tris-HCl, 0.15 M KCl) was added, and the samples were kept on ice. The fluorescent probes were then added, and the samples were incubated on ice in the dark to allow for probe incorporation. Immediately after, the samples were incubated for 10 min under standard culture conditions. Subsequent analysis of probe mobility in the lipid membrane was performed by measuring fluorescence anisotropy using a Cary Eclipse fluorescence spectrophotometer. The specific excitation and emission wavelengths used were 355/516 nm for DAUDA and 355/430 nm for TMA-DPH.

### 8.3. Measurement of Cleaved PARP Levels

To evaluate the impact of the compounds on apoptosis, the levels of cleaved poly (ADP-ribose) polymerase (PARP) were quantified using Human PARP (Cleaved) [214/215] ELISA Kit (Thermo Fisher, Cat# KHO0741). For this assay, cells were seeded into 35 mm dishes at a density of 1 × 10^6^ cells per dish and cultured in the appropriate medium for 24 h to ensure they were in the logarithmic growth phase. Following this, the test compounds were introduced at their respective IC_50_ concentrations, and the cultures were incubated for an additional 24 h.

After the incubation period, the culture medium was removed, and the cells were washed with a phosphate-buffered saline (PBS) solution. The cells were then detached from the dish using trypsinization. The resulting cell pellets were harvested and subsequently lysed in a RIPA buffer (50 mM Tris-HCL, pH 8.0, supplemented with 150 mM sodium chloride, 1.0% Igepal CA-630, 0.5% sodium deoxychlorate, and 0.1% sodium dodecyl sulfate). A protease inhibitor, phenylmethylsulfonyl fluoride, was also included in the lysis buffer to prevent protein degradation. Before measurement of cleaved PARP levels, protein concentration in cells lysates was determined using the method of Lowry et al. [110]. The concentration of cleaved PARP was determined using a specific ELISA kit, the PARP Cleaved [214/215] ELISA kit (Invitrogen, Waltham, MA, USA), in strict accordance with the manufacturer’s protocol. The absorbance of the resulting PARP degradation was measured using a microplate reader (Biotek Power Wave HT, Biotek Instruments, Winooski, VT, USA).

### 8.4. Caspase-3 and -9 Concentration Measurement

In order to determine the apoptotic pathways of cancer cells incubated with the tested derivatives, the level of active caspase-3 cleaved at Asp175/Ser176 and caspase-9 in cell lysates was measured using active Caspase-3 ELISA Kit (Thermo Fisher, Cat# KHO1091) and Caspase-9 ELISA Kit (Thermo Fisher, Cat# BMS2025) following the manufacturer’s instructions. For this assay, cells were seeded into 35 mm dishes at a density of 1 × 10^6^ cells per dish and cultured in the appropriate medium for 24 h to ensure they were in the logarithmic growth phase. Following this, the test compounds were introduced at their respective IC_50_ concentrations, and the cultures were incubated for an additional 24 h.

After the incubation period, the culture medium was removed, and the cells were washed with a phosphate-buffered saline (PBS) solution. The cells were then detached from the dish using trypsinization. The resulting cell pellets were harvested and subsequently lysed in a RIPA buffer (50 mM Tris-HCL, pH 8.0, supplemented with 150 mM sodium chloride, 1.0% Igepal CA-630, 0.5% sodium deoxychlorate, and 0.1% sodium dodecyl sulfate). A protease inhibitor, phenylmethylsulfonyl fluoride, was also included in the lysis buffer to prevent protein degradation. Before measurement of cleaved PARP levels, protein concentration in cells lysates was determined using the method of Lowry et al. [110].

### 8.5. Analysis of the MAPK Signaling Pathway-Measurement of Phosphorylated ERK 1/2, p38 and JNK 1/2

The mitogen-activated protein kinase (MAPK) pathway is a cascade of proteins that transmit signals from the outside of the cell to the nucleus, controlling fundamental cellular processes such as cell division, differentiation, and apoptosis. In cancer, these pathways are often overactivated or dysfunctional, leading to uncontrolled cell division, resistance to apoptosis, and tumor growth, making them an important therapeutic target.

In order to determine the involvement of mitogen-activated kinases (MAPK pathway) in the proliferation of cancer cells, analyses of the level of ERK 1/2, p38 MAPK and JNK 1/2 in cell lysates was measured using Multispecies MAPK Family Activation InstantOne^TM^ ELISA Kit (Thermo Fisher, Cat# 85-86195-11) following the manufacturer’s instructions. For this assay, cells were seeded into 35 mm dishes at a density of 1 × 10^6^ cells per dish and cultured in the appropriate medium for 24 h to ensure they were in the logarithmic growth phase. Following this, the test compounds were introduced at their respective IC_50_ concentrations, and the cultures were incubated for an additional 24 h.

After the incubation period, the culture medium was removed, and the cells were washed with a phosphate-buffered saline (PBS) solution. The cells were then detached from the dish using trypsinization. The resulting cell pellets were harvested and subsequently lysed in a RIPA buffer (50 mM Tris-HCL, pH 8.0, supplemented with 150 mM sodium chloride, 1.0% Igepal CA-630, 0.5% sodium deoxychlorate, and 0.1% sodium dodecyl sulfate). A protease inhibitor, phenylmethylsulfonyl fluoride, was also included in the lysis buffer to prevent protein degradation. Before measurement of cleaved PARP levels, protein concentration in cells lysates was determined using the method of Lowry et al. [110].

### 8.6. Determining the Level of Human Nrf2 in Cancer Cells

Focusing on the prooxidant properties of the tested derivatives and the participation of reactive oxygen species in shaping the overall antiproliferative activity of the analyzed compounds, the activity of nuclear factor erythroid 2 (NRF2) was measured.

To assess changes in NRF2 levels in cancer cells exposed to the tested derivatives, the Human NRF2 ELISA Kit (Thermo Fisher, Cat# EH348RB) was used. For this assay, cells were seeded into 35 mm dishes at a density of 1 × 10^6^ cells per dish and cultured in the appropriate medium for 24 h to ensure they were in the logarithmic growth phase. Following this, the test compounds were introduced at their respective IC_50_ concentrations, and the cultures were incubated for an additional 24 h.

After the incubation period, the culture medium was removed, and the cells were washed with a phosphate-buffered saline (PBS) solution. The cells were then detached from the dish using trypsinization. The resulting cell pellets were harvested and subsequently lysed in a RIPA buffer (50 mM Tris-HCL, pH 8.0, supplemented with 150 mM sodium chloride, 1.0% Igepal CA-630, 0.5% sodium deoxychlorate, and 0.1% sodium dodecyl sulfate). A protease inhibitor, phenylmethylsulfonyl fluoride, was also included in the lysis buffer to prevent protein degradation. Before measurement of cleaved PARP levels, protein concentration in cells lysates was determined using the method of Lowry et al. [110].

## 9. Statistical Significance Analysis and Figures

The statistical significance of the results was assessed using the raphPad Prims 9.0 (GraphPad, San Diego, CA, USA). One-way ANOVA analysis of variance and Tukey’s post hoc test for multiple comparisons were used. The significance level of the results was *p* < 0.05. The arithmetic mean and standard deviation were calculated using Microsoft Excel (Microsoft Office 2019, Microsoft Corporation, Redmond, WA, USA). The results of the study were presented in the form of arithmetic mean and standard deviation (SD).

**Figure 8:**
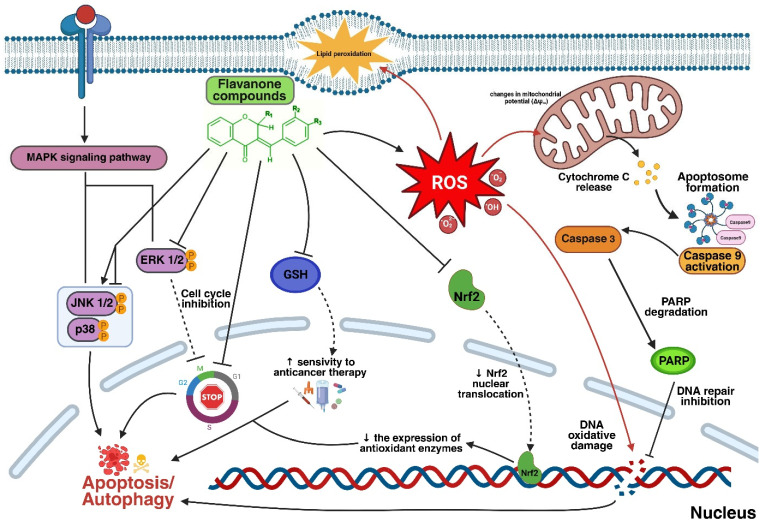
The proposed mechanism of action for the studied flavanone/chromanone derivatives against colon cancer cells is derived from our research, demonstrating that their anticancer activity is achieved through the modulation of multiple signaling pathways primarily involved in two areas: the induction of proapoptotic and antiproliferative events and the modulation of oxidative stress. The generation of ROS is crucial for the action of these derivatives in cancer cells, leading to severe molecular consequences, including lipid peroxidation and DNA damage. The observed mitochondrial damage (manifested as changes in the mitochondrial membrane potential), serves as a clear signal to initiate the intrinsic PCD pathway, resulting in the activation of caspase-9 and caspase-3. Degradation of PARP involved in these pathways simultaneously limits the cancer cell’s ability to repair the DNA damage incurred. Crucially, concurrent with increased ROS levels in cancer cells, the flavanone derivatives effectively reduce the activity of cellular defense mechanisms by inhibiting the transcription factor Nrf2 and decreasing the activity of the key antioxidant GSH. This dual decrease in Nrf2 and GSH activity likely translates into decreased expression of genes encoding antioxidant enzymes, thereby increasing the sensitivity of cancer cells to anticancer therapies. The pronounced blockade of the cell cycle in the G2/M phase may be attributed to the modulation of key kinases within the MAPK pathway. A significant inhibition of ERK1/2 was observed, which undoubtedly contributes to the antiproliferative activity. However, the effects on JNK1/2 and p38 are not unambiguous, as both increases and decreases in the activity of these kinases were observed, depending on the specific cell line. The black arrow (→) indicates activation/increase in expression of a specific pathway/gene. The red arrow (→) indicates a direct effect on a given cell structure. Inhibition of pathways/genes is indicated as (⊣).

BioRender was used to create Figure 8 with a license purchased by the Department of Cancer Biology and Epigenetics of the University of Lodz.

## Data Availability

The original contributions presented in this study are included in the article. Further inquiries can be directed to the corresponding authors.
